# Impact of a Social Marketing Intervention on General Practitioners’ Antibiotic Prescribing Practices for Acute Respiratory Tract Complaints in Malta

**DOI:** 10.3390/antibiotics10040371

**Published:** 2021-03-31

**Authors:** Anna Machowska, Gaetano Marrone, Peter Saliba-Gustafsson, Michael A. Borg, Erika A. Saliba-Gustafsson, Cecilia Stålsby Lundborg

**Affiliations:** 1 Department of Global Public Health, Health Systems and Policy: Improving Use of Medicines, Karolinska Institutet, 171 77 Stockholm, Sweden; anna.machowska@ki.se (A.M.); gaetano.marrone@ki.se (G.M.); cecilia.stalsby.lundborg@ki.se (C.S.L.); 2 Center for Molecular Medicine at BioClinicum, Cardiovascular Medicine Unit, Department of Medicine, Karolinska Institutet, Karolinska University Hospital Solna, 171 76 Stockholm, Sweden; peter.gustafsson.1@ki.se; 3 Division of Cardiovascular Medicine, Stanford University School of Medicine, Palo Alto, CA 94305, USA; 4 Department of Infection Prevention and Control, Mater Dei Hospital, MSD 2090 Msida, Malta; michael.a.borg@gov.mt; 5 Faculty of Medicine and Surgery, University of Malta, MSD 2090 Msida, Malta; 6 Division of Primary Care and Population Health, Stanford University School of Medicine, Stanford, CA 94305, USA

**Keywords:** antibiotics, antibiotic use, behaviour change, surveillance, primary care, general practitioners, respiratory tract infections, culture, Malta

## Abstract

*Introduction***:** Antibiotics are commonly prescribed in primary care for acute respiratory tract complaints (aRTCs), often inappropriately. Social marketing interventions could improve prescribing in such settings. We evaluate the impact of a social marketing intervention on general practitioners’ (GPs’) antibiotic prescribing for aRTCs in Malta. *Methods***:** Changes in GPs’ antibiotic prescribing were monitored over two surveillance periods between 2015 and 2018. Primary outcome: change in antibiotic prescription for aRTCs. Secondary outcomes: change in antibiotic prescription: (i) for immediate use, (ii) for delayed antibiotic prescription, (iii) by diagnosis, and (iv) by antibiotic class. Data were analysed using clustered analysis and interrupted time series analysis (ITSA). *Results***:** Of 33 participating GPs, 18 successfully completed the study. Although clustered analyses showed a significant 3% decrease in overall antibiotic prescription (*p =* 0.024), ITSA showed no significant change overall (*p =* 0.264). Antibiotic prescription decreased significantly for the common cold (*p* < 0.001), otitis media (*p =* 0.044), and sinusitis (*p =* 0.004), but increased for pharyngitis (*p =* 0.015). *Conclusions***:** The intervention resulted in modest improvements in GPs’ antibiotic prescribing. A more top-down approach will likely be required for future initiatives to be successful in this setting, focusing on diagnostic and prescribing support like rapid diagnostic testing, prescribing guidelines, and standardised delayed antibiotic prescriptions.

## 1. Introduction

Antibiotics are cost-effective, life-saving medicines that have contributed to an extended life expectancy [1]. The rapid escalation of antibiotic resistance (ABR) has however compromised their efficacy, posing a major threat to global health and sustainable development [2]. Infections caused by resistant microorganisms take longer to resolve, put patients at higher risk of poor clinical outcomes, and increase healthcare resource utilisation [3]. Concern is therefore growing that therapeutic options will become increasingly limited if ABR rates continue to rise.

Antibiotic consumption is a key driver of ABR [4], particularly antibiotic overuse and misuse through, for example, over-prescribing, inappropriate drug choice and treatment regimens and unregulated consumer access to antibiotics [5]. In Europe, although the overall antibiotic consumption trend has been stable, it varies by country, with significantly higher use in southern and eastern European countries [6]. Most antibiotics are prescribed for systemic use in primary care, with respiratory tract infections (RTIs) being the most common diagnoses [7]. Although antibiotics are necessary to effectively treat select RTIs (e.g., community-acquired pneumonia), most RTIs are viral and self-limiting, for which antibiotics have no clinical benefit. Bacterial RTIs are also often self-limiting, rendering the effects of antibiotics modest, sometimes negligible [8]. Despite this, antibiotics are estimated to be unnecessarily or inappropriately prescribed in over 50% of RTI cases [7,9,10,11]. Consequently, resistance rates in southern European countries have reached alarming levels [12].

Malta, a southern European country, has had consistently high antibiotic consumption rates [6,13,14,15,16]. In a 2018 Eurobarometer survey, 42% of Maltese respondents confirmed taking at least one antibiotic course during the previous 12 months, the top two reasons being sore throat and the flu [13]. Although surveys show that antibiotic consumption has decreased by 6% since 2016 [14], it remains the second highest in Europe [13]. Notably, the majority (96%) of antibiotics consumed are a result of a doctor’s prescription [13]. Addressing the diagnosis and treatment of RTIs is therefore paramount, and general practitioners (GPs) are an important target group.

Improving the quality of antibiotic prescribing in primary care is cornerstone to control ABR. Changing GPs’ prescribing behaviour could have far-reaching consequences, impacting not only their practices, but also patients’ beliefs and attitudes towards antibiotics. However, changing prescribing behaviour is complex and largely influenced by context and cultural norms [17]. Educational interventions may significantly reduce unnecessary prescribing [18], although multifaceted interventions targeting healthcare providers are more likely effective [19,20]. Social marketing (SM) is one approach that can induce sustained behaviour change [21]. This technique applies marketing theories to promote voluntary behaviour change [22] and has been widely applied in infection prevention and control [23]. Yet, there is a paucity of studies that describe [24] and use and evaluate [25] this approach to change antibiotic prescribing specifically. In this study, we describe the implementation of a SM intervention and evaluate its impact on GPs’ antibiotic prescribing for acute respiratory tract complaints (aRTCs) in Malta.

## 2. Results

### 2.1. GP Participation and Reported aRTC Cases

**Figure 1:**
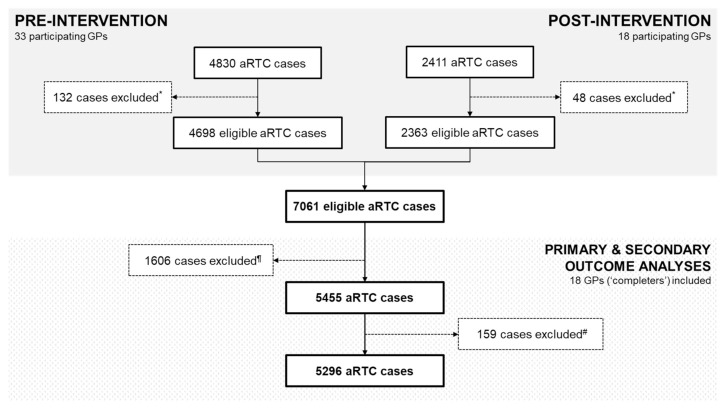
Flow chart of the total number of cases reported at pre- and post-intervention and those considered eligible for analysis. **NOTES:** * cases who did not meet inclusion criteria were excluded; ^¶^ cases reported by ‘non-completers’, i.e., GPs who did not participate in the post-intervention surveillance, were excluded from all primary and secondary outcome analyses; ^#^ for analysis of antibiotic prescribing by diagnosis, cases diagnosed with secondary diagnoses were excluded.

During the pre-intervention phase, 4830 aRTC cases were reported by 33 GPs. Of these, 132 cases did not meet inclusion criteria, resulting in 4698 eligible aRTC cases. During the post-intervention phase, 2411 aRTC cases were reported by 18 GPs. Of these, 48 cases did not meet inclusion criteria, resulting in 2363 eligible aRTC cases. Ultimately, 7061 eligible aRTC cases were included. Fifteen GPs did not complete the study and are henceforth referred to as ‘non-completers’. Consequently, for primary and secondary outcome analyses, their cases (*n =* 1606) were excluded, rendering 5455 eligible aRTC cases. For analysis of antibiotic prescribing by diagnosis, a further 159 patients with secondary diagnoses were excluded (Figure 1).

### 2.2. GP Characteristics

GP characteristics were similar pre- and post-intervention (Table 1). However, compared to ‘completers’, ‘non-completers’ were older on average (*p =* 0.025), had more years of clinical practice (*p =* 0.004) and higher pre-intervention antibiotic prescribing rates (*p =* 0.000) (Table 2).

### 2.3. Change in Overall Antibiotic Prescription Rates Pre- and Post-Intervention among 33 GPs (n = 7061 Eligible aRTC Cases)

Overall antibiotic prescription for aRTC patients decreased significantly post-intervention from 2152 (45.8%) to 890 (37.7%) (*p =* 0.016). Of these, 343 (15.9%) and 285 (32.0%) prescriptions were delayed antibiotic prescriptions (DAPs) at pre- and post-intervention respectively, resulting in a significant increase in the proportion of DAPs prescribed post-intervention (*p =* 0.001). The rest were antibiotics prescribed for immediate use. This included 1809 (84.1%) before and 605 (68.0%) after the intervention; also a significant decrease post-intervention (*p =* 0.001).

### 2.4. Impact of the SM Intervention on Antibiotic Prescription Rates among 18 GP ‘Completers’ (n = 5455 Eligible aRTC Cases)

#### 2.4.1. Primary Outcome Analysis

**Figure 2:**
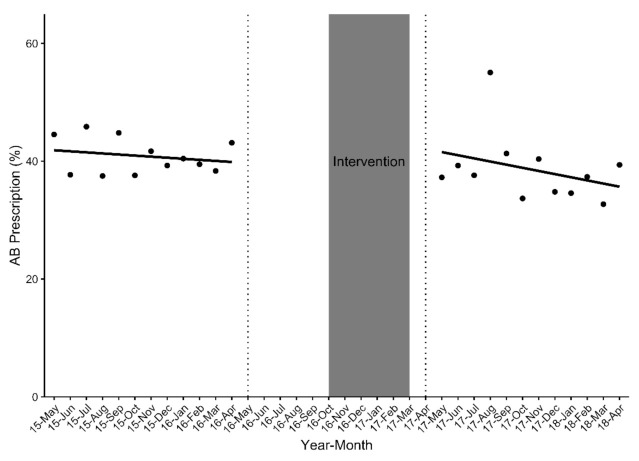
Change in the overall proportion of antibiotics prescribed (% AB prescription) to patients with acute respiratory tract complaints pre- and post-intervention (*n =* 5455 eligible aRTC cases).

When restricting analysis to the 18 GP ‘completers’, before-and-after clustered analysis showed that less patients received antibiotics post-intervention (*n =* 1260 (40.8%) vs *n =* 890 (37.7%)) (*p =* 0.024). Interrupted time series analysis (ITSA) however showed no significant change in antibiotic prescription, both directly after the intervention and 1 year later (Table 3). Antibiotic use decreased non-significantly by 0.18% prior to the intervention (*p =* 0.264). Immediately post-intervention, there was a non-significant increase in antibiotic prescription of 1.86% (*p =* 0.633). This was followed by a non-significant decrease in the antibiotic prescription trend (relative to the pre-intervention trend) of 0.35% per month (*p =* 0.371). Following the intervention, antibiotic prescription decreased non-significantly at a rate of 0.53% per month (*p =* 0.182) (Figure 2).

#### 2.4.2. Secondary Outcome Analysis: Antibiotic Prescription for Immediate Use among aRTC Cases Who Received an Antibiotic Prescription (*n* = 2150 Eligible aRTC Cases)

**Figure 3:**
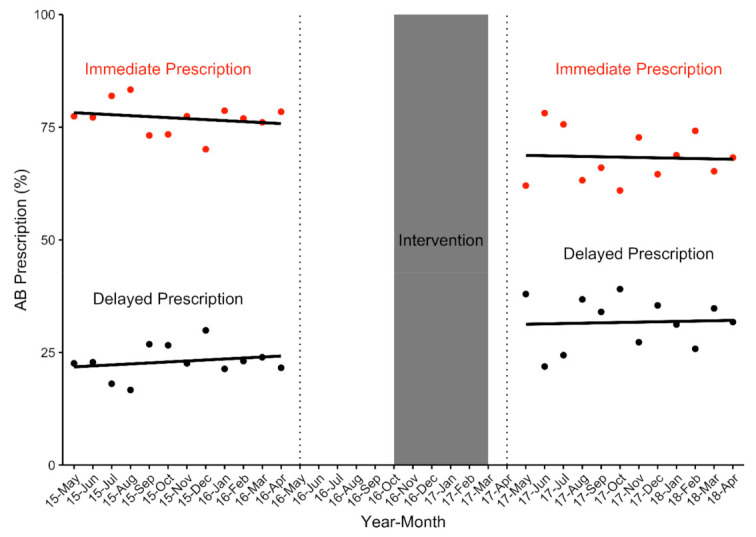
Change in the proportion of antibiotics (% AB prescription) issued to patients with acute respiratory tract complaints for delayed and immediate use pre- and post-intervention (*n =* 2150 eligible aRTC cases).

Before-and-after clustered analysis showed that significantly less patients were given a prescription for immediate use after the intervention (*n =* 967 (76.8%) vs *n =* 605 (68.0%)) (*p =* 0.002). ITSA however showed no significant change in antibiotic prescription for immediate use directly after or 1-year post-intervention (Table 3). Antibiotic prescription for immediate use decreased non-significantly by 0.22% prior to the intervention (*p =* 0.349). Immediately post-intervention, there was a non-significant decrease in antibiotic prescription of 6.84% (*p =* 0.126). This was followed by a non-significant increase in the antibiotic prescription trend of antibiotics for immediate use (relative to the pre-intervention trend) of 0.14% per month (*p =* 0.771). Following the intervention, antibiotic prescription for immediate use decreased non-significantly at a rate of 0.08% per month (*p =* 0.867) (Figure 3).

#### 2.4.3. Secondary Outcome Analysis: DAP among aRTC Cases who Received an Antibiotic Prescription (*n* = 2150 Eligible aRTC Cases)

Significantly more DAPs were prescribed post-intervention (*n =* 293 (23.3%) vs 285 (32.0%)) (*p =* 0.002) when analysed using before-and-after clustered analysis. Once again, ITSA showed no significant change in DAP directly after or 1-year post-intervention (Table 3). DAP increased non-significantly by 0.22% prior to the intervention (*p =* 0.349). Immediately post-intervention, there was a non-significant increase in DAP of 6.84% (*p =* 0.126). This was followed by a non-significant decrease in the DAP trend (relative to the pre-intervention trend) of 0.14% per month (*p =* 0.771). Following the intervention, DAP increased non-significantly at a rate of 0.08% per month (*p =* 0.867) (Figure 3).

#### 2.4.4. Secondary Outcome Analysis: Antibiotic Prescribing by Diagnosis among Eligible aRTC Cases Reported by GP ‘Completers’ (*n =* 5296 Eligible aRTC Cases)

The diagnoses that consistently received the highest proportion of antibiotic treatment both pre- and post-intervention were, tonsillitis, bronchitis and otitis media. Diagnoses like the common cold and influenza, both viral infections, received antibiotic prescriptions during both phases, although prescribing decreased significantly post-intervention for the common cold (*p* < 0.001). A significant decrease in antibiotic prescribing was also observed for otitis media (*p =* 0.044) and sinusitis (*p =* 0.004). Notably, antibiotic prescribing for pharyngitis increased significantly post-intervention (*p =* 0.015) (Table 4).

Prescription of β-lactam antibacterials, penicillins (J01C) decreased significantly post-intervention for the common cold (*p =* 0.021). Concurrently, use of 2 other antibiotic classes decreased for this indication. Prescription of other β-lactam antibacterials (J01D) decreased significantly post-intervention for sinusitis (*p =* 0.001) and bronchitis (*p =* 0.040). Although macrolide use decreased significantly for influenza (*p =* 0.032), its use increased significantly for pharyngitis (*p =* 0.046) (Table 5).

#### 2.4.5. Secondary Outcome Analysis: Antibiotic Prescribing in the Three Most Commonly Prescribed Antibiotic Classes (*n =* 2150 Eligible aRTC Cases)

The most commonly prescribed antibiotic class (both pre- and post-intervention) was β-lactam antibacterials, penicillins (J01C), with co-amoxiclav constituting the majority of prescriptions. Macrolides (J01F) were the second most commonly prescribed antibiotic class, with clarithromycin and azithromycin being the most favoured antibiotics. Other β-lactam antibacterials (J01D) were also among the top 3 most commonly prescribed antibiotics pre- and post-intervention although less often prescribed. Clustered analysis showed that prescription of other β-lactam antibacterials (J01D) decreased significantly post-intervention from 19.2% to 13.1% (*p* < 0.001). Prescription of tetracyclines (J01A) and β-lactam antibacterials, penicillins (J01C) however, increased significantly post-intervention from 0.7% to 2.2% (*p =* 0.002) and 46.8% to 53.3% (*p =* 0.032), respectively (Table 6).

## 3. Discussion

### 3.1. Summary of Findings

To our knowledge, this is the second study conducted in a Mediterranean setting that aims to rigorously evaluate the impact of a SM intervention on GPs’ antibiotic prescribing for respiratory tract complaints. Before-and-after clustered analysis showed that the intervention resulted in a significant 3% decrease in overall antibiotic prescription, a 9% decrease in prescription for immediate use, and a 9% increase in DAP. Although ITSA showed no significant impact on GPs’ overall antibiotic prescribing trends over time, clustered analysis demonstrated significant decreases in antibiotic prescribing for some diagnoses post-intervention. Notably however, despite emphasis on the repercussions of unnecessary antibiotic prescribing for pharyngitis during educational sessions, and distribution of national antibiotic prescribing guidelines, antibiotic prescribing for pharyngitis increased significantly post-intervention. GPs also continued to prescribe antibiotics unnecessarily for the common cold. Finally, although antibiotic prescription of other β-lactam antibacterials (J01D) decreased significantly post-intervention for sinusitis and bronchitis, macrolide (J01F) use for pharyngitis increased significantly.

### 3.2. Impact of Multifaceted Interventions on Antibiotic Prescribing

Multifaceted interventions coupled with behaviour change theories can successfully improve antibiotic prescribing [26]. In our study, all GPs were exposed to a complex, multifaceted SM intervention, which utilises behavioural and social science principles, and commercial marketing to influence positive behaviour [21,27]. Following the SM intervention, the proportion of antibiotic prescriptions decreased (overall and for immediate use), whilst DAP increased. ITSA suggests that the intervention did not significantly change GPs’ antibiotic prescribing practices. Given the paucity of studies in this area, further investigation into whether SM techniques can sustain behaviour change in similar settings is warranted. A similar study conducted in northern Italy that also utilized SM techniques to influence outpatient antibiotic prescribing, succeeded in reducing antibiotic prescribing significantly by 4.3%, suggesting that SM approaches could lead to successful and clinically meaningful changes in antibiotic prescribing practices [25]. Other studies have also reported improvements in antibiotic prescribing following behaviour change interventions, albeit not SM. A cluster RCT carried out in 47 primary practices in Boston (MA, USA) evaluating the impact of three behavioural interventions implemented alone or in combination (suggested alternative treatment, accountable justification, and peer comparison), showed that accountable justification and peer comparison decreased inappropriate antibiotic prescribing for acute RTIs [28]. Similarly, a Dutch pragmatic RCT evaluating the impact of a multifaceted intervention (physician education and audit/feedback) on GPs’ antibiotic prescribing also showed a significant decrease in antibiotic prescribing for RTIs and prescribing of non-first-choice antibiotics for RTIs [7]. Whilst our intervention neither included audit/feedback nor peer comparison due to limited resources, GPs received feedback reports. Although this passive information provision could have impacted GPs’ antibiotic prescribing, it did not appear to have an impact in our study. Another similar study, the Happy Audit, implemented a multifaceted intervention (clinical guidelines, waiting room posters, patient brochures, point-of-care tests (POCTs), and training), with GPs from Argentina, Denmark, Lithuania, Russia, Spain, and Sweden. Although GPs’ antibiotic prescribing rates varied across countries, the intervention decreased antibiotic prescribing in Baltic and Hispano-America countries, but not in the Nordics [29].

### 3.3. Culture and Behaviour Change

Changing clinicians’ antibiotic prescribing behaviour is challenging, particularly since prescribing culture is shaped by numerous factors including clinical, cultural, economic, and social factors [26,30]. Malta has the second highest uncertainty avoidance score in Europe [31], a cultural dimension that has consistently been reported as a potent driver of excessive or unnecessary antibiotic use [32,33,34]. In these cultures, changing behaviour is particularly challenging as people are generally more resistant to change [33]. Uncertain and ambiguous situations tend to make people feel threatened and anxious, and are typically not well-tolerated. In such a context, doctors may feel compelled to act promptly when faced with uncertain clinical presentations. Rather than employing a wait and see approach [32] they may prescribe antibiotics immediately, “just in case”, to provide a subconscious reassurance of certainty to both the patient and prescriber [33,35], even in situations where antibiotics provide no clinical benefit. This is evident from our recent study where GPs explained that they occasionally prescribe antibiotics for sore throat because they are concerned about a possible bacterial aetiology or development of a secondary bacterial infection [36]. Patient expectations and demand can further amplify the problem. In fact, patient demand for antibiotics is correlated with inappropriate antibiotic prescribing [15,37,38,39].

In such cultural backgrounds, it is more likely that broad-spectrum antibiotics are used [40] as shown in our study [15]. Despite educational sessions on the repercussions of unnecessary broad-spectrum antibiotic prescribing and encouragement to opt for narrow-spectrum agents instead, GPs continued to prescribe broad-spectrum antibacterials post-intervention. Of particular concern is the persistent prescription of antibiotics for viral infections such as the common cold and influenza, as well as the significant increase in antibiotic prescribing for pharyngitis post-intervention. Specifically, the increase in macrolide prescriptions, which is not indicated as first-line treatment for pharyngitis, is worth noting, particularly since macrolides are key drivers of macrolide-resistance in streptococci [4,41]. Indeed, antibiotics are often not required to treat acute pharyngitis. Pharyngitis caused by group A β-haemolytic streptococcus is only found in about 10-15% of adult infections [42,43]. Importantly, studies show that, 85% of acute pharyngitis patients are completely symptom free within a week, irrespective of whether they receive antibiotics or not [44], and the risk of suffering serious complications is uncommon [45,46,47].

### 3.4. Rapid Point-of-Care Tests

Rapid POCTs could help decrease antibiotic prescribing rates by reducing GP uncertainty. They could also lessen the risk that GPs succumb to patient pressure by supporting their decision not to prescribe antibiotics [48,49]. Indeed, POCTs can reduce antibiotic prescribing in patients who request an antibiotic [49]. Rapid POCTs however, do not guarantee appropriate prescribing practices [50]. In fact, a Spanish study showed that antibiotics were prescribed in more than 30% of cases with negative rapid antigen detection tests for acute pharyngitis [51]. Unsurprisingly, Spain, like Malta, exhibits rather high uncertainty avoidance scores. Rapid POCTs should therefore be used when justified and according to established guidelines, such as Centor criteria.

Rapid, low-cost diagnostic tests are still largely unavailable in Malta despite their potential in reducing unnecessary antibiotic prescribing [50,51]. Future interventions in this context should focus on addressing GP uncertainty without compromising patient satisfaction. Given the success of POCTs in similar contexts [49], we believe that if implemented correctly, POCTs could improve antibiotic prescribing in this setting. However, POCTs will pose additional costs, which could be a deterrent in the Maltese system as they would add to the cost of the GP consultation. Nevertheless, they would provide GPs and patients alike with a greater sense of security, particularly in cases such as pharyngitis. Any successful roll-out of POCTs will need to be cognizant of barriers to adoption and avoid introducing new elements of uncertainty. Coupled with GP training to encourage acceptability, and patient-communication training, we believe that this approach has great potential in our setting, especially since 90% of the Maltese consider doctors their most trustworthy source of information [13]. An alternative could be for these tests to be undertaken in pharmacies with patients testing negative offered symptomatic relief. This would be economically advantageous to the patient as it would save the cost of a consultation in most cases and would be considered cost-effective.

### 3.5. Delayed Antibiotic Prescription

DAP can also address uncertainty [52,53,54,55] and effectively reduce antibiotic consumption for RTIs, with little to no impact on increased risk for complications, illness duration, and time to symptom resolution [56,57,58,59,60]. The utilisation of DAPs can be underpinned by cultural factors and prescriber attitudes. A European-wide study showed that GPs’ attitudes towards DAPs for acute cough varies and appears to be less commonly used in southern European countries [61]. In our previous study, GPs were largely positive towards DAPs, although not all supported the strategy; some preferred a wait-and-see approach with in-person follow-up [35]. They also preferred to selectively practice DAP with patients they trusted or who they believed had a certain level of knowledge and understanding [35]. In the present study, it is encouraging to see that adoption of DAP increased post-intervention, suggesting that continuing to encourage the use of DAP can potentially promote more appropriate antibiotic use in Malta. Building the necessary infrastructure to determine the utilisation of such prescriptions would allow us to better evaluate the success of this intervention. Unfortunately, due to resource and structural limitations in this setting, it was not possible to track whether DAPs issued were indeed dispensed (and subsequently consumed).

### 3.6. Academic Detailing

In our study, older and more experienced GPs with higher antibiotic prescribing rates, dropped out of the study during or shortly after the intervention. GP age (>60 years) was previously identified as a significant predictor of higher antibiotic prescribing [38]. It is therefore paramount to tailor future interventions to engage this subset of GPs in behaviour change initiatives. Academic detailing is one strategy that could be used; it has been shown to positively impact antibiotic prescribing [18] and increase guideline-concordant antibiotic use for RTIs [62]. Much like strategies used by pharmaceutical representatives [63], physicians receive one-to-one educational visits by trained healthcare professionals within their own professional environment [64]. Through this feedback, GPs can evaluate their own prescribing practices and compare it to that of others, and also receive evidence-based guidance and recommendations on appropriate antibiotic use.

In Malta, GPs typically lack information and therefore appreciate information provided to them by pharmaceutical representatives, although they acknowledge that information may be biased [36]. Academic detailing may therefore help bridge the need for up-to-date and unbiased knowledge. In our setting however, this strategy would not only require human and financial resources, but also the necessary IT infrastructure to record antibiotic prescribing data, which is currently lacking.

### 3.7. Strengths and Limitations

To our knowledge this is the second study that evaluates the impact of a SM intervention to change GPs’ antibiotic prescribing in a Mediterranean setting. Moreover, it is the first time that a rigorous study design has been applied in Malta to monitor and change antibiotic prescribing patterns for aRTCs in primary care. The study involved detailed antibiotic prescribing surveillance conducted over 24 months, generating unique data.

Every study, however, needs to be seen in the context of its limitations. Firstly, during or shortly after the intervention, 15 GPs who had significantly higher antibiotic prescribing rates pre-intervention dropped out of the study and did not contribute to our post-intervention data collection. This reduced our sample size and could explain a lack of significant results when using ITSA. It also makes interpretation of the intervention results challenging as it appears that GPs who remained in the study were more willing and motivated to change their antibiotic prescribing practices. Nevertheless, despite GPs’ higher motivation and engagement, only some improvement in their antibiotic prescribing practices was observed. Moreover, not all GPs participated in the intervention to the same extent. Their varying exposure could have inevitably impacted their antibiotic prescribing post-intervention. Finally, due to the study’s small GP cohort and lack of national antibiotic prescribing data, it was neither possible to tailor intervention components to GPs’ individual stage of behaviour change, nor assign a control group for comparison. In addition, the study’s multifaceted design did not allow us to measure the effect of each individual component to determine which intervention had the largest impact on GPs’ antibiotic prescribing.

## 4. Material and Methods

### 4.1. Study Design, Setting and Participants

This 4-year quasi experimental study was conducted in primary care practices across Malta between 2014 and 2018 [65]. Briefly, between 2014 and 2016, qualitative and quantitative studies were conducted to inform the design, development, and implementation of a 6-month SM intervention implemented between October 2016 and March 2017. These included: (i) GP interviews; (ii) focus group discussions (FGDs) with GPs, pharmacists, and parents; (iii) antibiotic prescribing surveillance; and (iv) behaviour change GP questionnaires. Pre-intervention findings and relevant data collection tools have already been published elsewhere [15,35,36,38,65].

#### 4.1.1. Pre- and Post-Intervention Antibiotic Prescribing Surveillance

**Figure 4:**
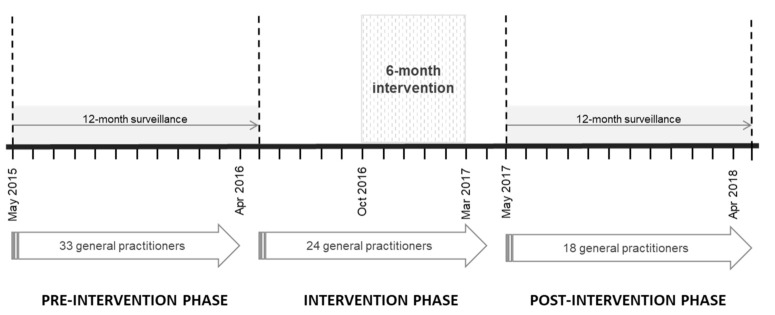
Surveillance and intervention timeline, including general practitioner participation at each phase.

In 2014, during the pre-intervention phase, 370 GPs and 34 GP trainees were invited to participate in a 1-year antibiotic prescribing surveillance study (May 2015 to April 2016). Ultimately, 30 GPs and 3 GP trainees participated. Following completion, GPs were invited to extend participation in an intervention and subsequent 1-year post-intervention surveillance study (May 2017 to April 2018). Although 24 GPs extended participation and partook in the intervention, 18 GPs reported cases during both the pre- and post-intervention phases, allowing for pre-/post-intervention comparative analyses (Figure 4).

Identical data collection methods and tools were used during the pre- and post-intervention phases [15]. GPs collected surveillance data for patients seen for aRTCs during a pre-determined 1-week period, each month, between May 2015 and April 2016 (pre-intervention), and May 2017 and April 2018 (post-intervention), with no substitutions. GPs were asked to complete forms during their first consultation with all patients suffering from any aRTC. GPs received 3 text message reminders during each surveillance week to prepare, initiate, and conclude data collection. Upon completion of each surveillance week, GPs sent completed forms to the local research team by postal mail in pre-paid envelopes. GPs were also called at most 4 times each surveillance year to address concerns and provide encouragement. Finally, GPs received 3-monthly individual- and aggregate-level feedback reports on their prescribing patterns and certificates of participation.

#### 4.1.2. Intervention Development, Design, and Delivery

The intervention comprised 5 components: (i) patient booklets; (ii) waiting room posters; (iii) national antibiotic prescribing guidelines; (iv) DAP pads; and (v) GP educational sessions. GPs received certificates for their participation, which they could have used for CME accreditation. Regular contact (via email and text message) was maintained with GPs throughout, to address queries, provide encouragement, and maintain engagement.

##### Patient Education Materials: Booklet and Posters

Patient booklets were developed based on needs identified during the pre-intervention phase and were informed by the team’s subject-area knowledge and evidence-informed guidelines. They were designed with a medical illustrator and underwent several revisions following team discussions and feedback from three medical professionals and five members of the public. The 6-page booklet included information in both national languages (i.e., English and Maltese) on the pathogenesis of RTIs, appropriate antibiotic use, the effect antibiotic use has on the body, and ABR (Suppl. File 1). A set of 4 waiting room posters (Suppl. File 2) were also developed by the research team. They were pre-tested during FGDs with three groups of parents (5–8 participants/group) and edited accordingly. A QR code was generated for both resources, directing readers to the Malta National Antibiotic Committee’s website [66] for more information on appropriate antibiotic use.

All participating GPs received a package of patient education materials, relative to their practice size. They were asked to put up posters at their clinics and encouraged to use and distribute patient booklets during consultations with aRTC patients who both received and did not receive an antibiotic prescription. GPs could request more materials if necessary.

##### Antibiotic Prescribing Support Tools

Both soft and hard copies of the updated national antibiotic guidelines were shared with GPs. GPs also received standardised DAP pads (Suppl. File 3) coupled with patient information on RTIs and appropriate antibiotic use in both national languages. GPs were instructed on how to use the pads during one-to-one meetings and could request more DAP pads if necessary. A memo was also circulated to all local pharmacies via the Malta Chamber of Pharmacists, informing pharmacists about the new DAPs.

##### GP Educational Sessions

GPs were invited to attend 4 interactive educational sessions, delivered both in-person and live online. Topics encompassed: (i) antibiotic use and resistance from a global and local perspective; (ii) introduction to the latest national antibiotic prescribing guidelines; (iii) principles of prudent antibiotic prescribing; and (iv) patient communication skills training. The 2-h sessions were held by local experts on antibiotic use and resistance, antibiotic pharmacology, and patient communication, on weekdays after 8pm. All sessions were recorded, allowing GPs to view the sessions at their own convenience via an online educational platform.

##### Intervention Delivery

Key process indicators for all five intervention components are presented in Table 7.

### 4.2. Data Analysis

#### 4.2.1. Inclusion and Exclusion Criteria

All patients suffering from aRTCs (defined as lower and upper RTIs, allergies and exacerbations of COPD/asthma/bronchitis) were considered cases. Cases reported outside the pre-determined surveillance weeks, who received non-oral antibiotics, had incomplete reporting sheets, were diagnosed with pneumonia, or follow-up cases, were not included. Further, all cases reported by the 15 GP ‘non-completers’, were excluded from all primary and secondary outcome analyses to enable pre- and post-intervention comparison. To analyse the change in prescription of immediate versus delayed antibiotics, and antibiotic class pre- and post-intervention, analysis was further restricted to patients who received an antibiotic prescription. Finally, to analyse the change in antibiotic prescribing by diagnosis, patients who received any secondary diagnoses were excluded.

#### 4.2.2. Statistical Analyses

The primary outcome of interest was change in antibiotic prescription (defined as an antibiotic prescription-immediate or delayed-of oral antibiotics issued for an aRTC during an in-person consultation, irrespective of the number of antibiotics given) among GP ‘completers’. Secondary outcomes included change in: (i) the proportion of antibiotic prescriptions for immediate use; (ii) the proportion of DAPs; (iii) antibiotic prescribing by diagnosis; and (iv) antibiotic prescribing by antibiotic class.

The WHO’s 2017 Anatomical Therapeutic Chemical (ATC) classification system was used to classify antibiotics [67]. Descriptive statistics were used to present GPs’ characteristics and antibiotic prescribing practices. Continuous variables were expressed as median and IQR, and categorical variables as frequencies and percentages. To compare GPs’ antibiotic prescription rates pre- and post-intervention, non-parametric Mann-Whitney U tests were used to compare differences in medians of continuous variables, and Pearson’s chi-squared tests were used for categorical variables. In cases where expected values were <5, Fisher’s Exact test was used. To control for clustering at GP level, population-averaged models using generalised estimating equations were used to compare antibiotic prescription pre- and post-intervention. Univariable associations between antibiotic prescription rates and the phase of the study (pre- versus post-intervention), were assessed using unadjusted ORs and 95% CIs. ITSA was also used to evaluate the intervention’s overall impact by comparing the trend in the proportion of antibiotics prescribed (immediate and delayed) for aRTCs pre- and post-intervention. *p*-values < 0.05 were considered significant. Data were input in Excel^®^ 2010 (Microsoft^®^, Redmond, WA, USA) and statistical analyses were performed using STATA^®^ version 15.1 (College Station, TX, USA).

## 5. Conclusions

### Conclusions and Recommendations

In this novel study we show that a SM intervention can, to some extent, positively change GPs’ antibiotic prescribing for aRTCs. Our intervention utilised a voluntary behaviour change approach, yet despite being delivered to GPs who were already highly motivated to change, ITSA showed no significant impact on their antibiotic prescribing behaviour. In cultural backgrounds that are more resistant to change, competing drivers may be too strong, especially in a fully autonomous private GP set up like in Malta. Subsequently, a more coercive top-down approach may have had a greater impact. Moreover, the lack of a national antibiotic prescribing surveillance system (and consequent lack of accountability) poses limitations; its set-up needs urgent attention.

Several other actions should be taken to improve GPs’ antibiotic prescribing in Malta. Increasing the availability and use of rapid POCTs is highly recommended to provide GPs with a greater sense of clarity and certainty, particularly since antibiotic use for pharyngitis increased post-intervention. With strong political will and stakeholder engagement, we believe that POCTs can limit unnecessary antibiotic prescribing. We also believe that formalising and standardising DAP could help reduce unnecessary antibiotic consumption and change public perceptions about the need for immediate antibiotics for aRTCs. Academic detailing could also provide GPs with evidence-based information on guideline-concordant antibiotic use and give them the opportunity to reflect upon their own antibiotic prescribing. This strategy should be well-accepted in Malta as GPs tend to be rather receptive towards information shared to them by pharmaceutical representatives. Future studies should focus on changing antibiotic prescribing practices for specific indications such as tonsillitis, bronchitis, pharyngitis, common cold and influenza, to achieve better guideline-concordance and further reduce unnecessary and premature antibiotic prescribing for aRTCs.

## Figures and Tables

**Table 1:** Comparison of general practitioners’ characteristics: pre- versus post-intervention.

GP Characteristics	Pre-Intervention (*n =* 33)	Post-Intervention (*n =* 18)	*p*-Value
Age, median (IQR)	52 (42–57)	46 (41–53)	0.221
Sex, *n* Male/Female	24/9	13/5	0.969
Years of GP practice, median (IQR)	23 (16–29)	20 (15–24)	0.121
Type of practice, *n* ^#^ Group/Solo	15/18	10/8	0.490
Employment sector, *n* Public only Private only Public & private	11 20 2	8 9 1	0.791
Employment type, *n* Full-time/Part-time	22/11	14/4	0.527

**NOTES:** GP *=* general practitioner; ^#^ public health centres were defined as group practices.

**Table 2:** Comparison of general practitioners’ characteristics: ‘completers’ versus ‘non-completers’.

GP Characteristics	Completers (*n =* 18)	Non-Completers (*n =* 15)	*p*-Value
Age, median (IQR)	46 (41–53)	55 (48–59)	**0.025**
Sex, *n* Male/Female	13/5	11/4	1.000
Years of GP practice, median (IQR)	20 (15–24)	29 (26–33)	**0.004**
Type of practice, *n* ^#^ Group/Solo	10/8	5/10	0.172
Employment sector, *n* Public only Private only Public & private	8 9 1	3 11 1	0.407
Employment type, *n* Full-time/Part-time	14/4	8/7	0.163
Antibiotic prescription (pre-intervention) *, *n* (%)	1260 (40.8%)	892 (55.5%)	**0.000**

**NOTES:** GP *=* general practitioner; ^#^ public health centres were defined as group practices; * defined as an antibiotic prescription-immediate or delayed-of oral antibiotics issued for an acute respiratory tract complaint during an in-person consultation, irrespective of the number of antibiotics given. Statistically significant values are marked in bold.

**Table 3:** Evaluation of the change in antibiotic prescription overall, for immediate use and delayed use, using interrupted time series analysis.

Outcome	Baseline Indicator Estimate	Baseline Trend *	Short-Term Effect *	Long-Term Effect *	Post-Intervention Linear Trend *
	*n* (%) [95% CI]	_t	_x13	_x_t13	_b[_t]+_b[_x_t13]
Antibiotic prescription	1295 (41.87) [40.11–43.62]	−0.18 [−0.51–0.15]	1.86 [−6.16–9.89]	−0.35 [−1.16–0.45]	−0.53 [−1.34–0.27]
Immediate antibiotic prescription	985 (78.21) [74.22–82.19]	−0.22 [−0.70–0.26]	−6.84 [−15.77–2.09]	0.14 [−0.86–1.15]	−0.08 [−1.04–0.88]
Delayed antibiotic prescription	275 (21.79) [17.81–25.78]	0.22 [−0.26–0.70]	6.84 [−2.09–15.77]	−0.14 [−1.15–0.86]	0.08 [−0.88–1.04]

**NOTES:** * estimate [95% CI].

**Table 4:** Diagnosis-specific antibiotic prescribing (overall) for the most common diagnoses, pre- and post-intervention (*n =* 5296 eligible aRTC cases).

Diagnosis (*n*)	Antibiotic Prescriptions (Pre) *n*/N (%)		Antibiotic Prescriptions (Post) *n*/N (%)		OR	95% CI	*p*-Value
Tonsillitis (*n =* 453)	251/267	(94.0)	176/186	(94.6)	1.06	0.49–2.33	0.876
Bronchitis (*n =* 585)	267/314	(85.0)	216/271	(79.7)	0.71	0.48–1.05	0.084
Otitis media (*n =* 125)	69/75	(92.0)	39/50	(78.0)	0.38	0.15–0.97	**0.044**
Pharyngitis (*n =* 812)	208/417	(49.9)	220/395	(55.7)	1.41	1.07–1.86	**0.015**
Sinusitis (*n =* 342)	106/190	(55.8)	64/152	(42.1)	0.60	0.42–0.85	**0.004**
Exacerbation ^¶^ (*n =* 288)	87/180	(48.3)	38/106	(35.8)	0.65	0.42–1.00	0.051
Influenza (*n =* 231)	35/105	(33.3)	23/126	(18.3)	0.59	0.33–1.06	0.076
Allergy (*n =* 264)	12/163	(7.4)	10/101	(10.0)	1.53	0.48–4.93	0.472
Common cold (*n =* 1952)	110/1145	(9.6)	37/807	(4.6)	0.51	0.36–0.73	**0.000**

**NOTES:**^¶^ exacerbation of chronic obstructive pulmonary disease/asthma/bronchitis. Statistically significant values are marked in bold.

**Table 5:** Diagnosis-specific antibiotic prescribing for the three most commonly prescribed antibiotic classes, pre- and post-intervention (*n =* 5296 eligible aRTC cases).

Diagnoses (*n*)	J01C (Pre)		J01C (Post)		OR	95% CI	*p*-Value	J01D (Pre)			J01D (Post)		OR	95% CI	*p*-Value	J01F (Pre)		J01F (Post)		OR	95% CI	*p*-Value
	*n* (%)		*n* (%)					*n* (%)			*n* (%)					*n* (%)		*n* (%)				
Tonsillitis (*n =* 453)	151	(56.6)	120	(64.5)	1.43	0.97–2.09	0.067	45	(16.9)	25		(13.4)	0.70	0.41–1.17	0.173	55	(20.6)	31	(16.7)	0.78	0.48–1.27	0.315
Bronchitis (*n =* 585)	118	(37.6)	116	(42.8)	1.00	0.74–1.36	0.999	35	(11.1)	17		(6.3)	0.53	0.29–0.97	**0.040**	84	(26.8)	63	(23.2)	1.10	0.75–1.62	0.625
Otitis media (*n =* 125)	42	(56.0)	21	(42.0)	0.63	0.31–1.29	0.207	13	(17.3)	8		(16.0)	0.94	0.42–2.09	0.873	14	(18.7)	10	(20.0)	0.91	0.47–1.75	0.775
Pharyngitis (*n =* 812)	88	(21.1)	98	(24.8)	1.29	0.97–1.72	0.079	44	(10.6)	33		(8.4)	0.77	0.48–1.22	0.263	71	(17.0)	86	(21.8)	1.46	1.01–2.12	**0.046**
Sinusitis (*n =* 342)	37	(19.5)	33	(21.7)	1.42	0.88–2.30	0.154	31	(16.3)	6		(3.9)	0.16	0.06–0.46	**0.001**	34	(17.9)	19	(12.5)	0.70	0.39–1.24	0.221
Exacerbation ^¶^ (*n =* 288)	45	(25.0)	24	(22.6)	0.79	0.47–1.34	0.386	16	(8.9)	3		(2.8)	0.37	0.11–1.21	0.099	16	(8.9)	4	(3.8)	0.46	0.15–1.36	0.159
Influenza (*n =* 231)	8	(7.6)	10	(7.9)	1.07	0.40–2.88	0.895	7	(6.7)	5		(4.0)	0.65	0.15–2.93	0.582	19	(18.1)	6	(4.8)	0.27	0.08–0.89	**0.032**
Allergy (*n =* 264)	5	(3.1)	8	(7.9)	4.01	0.48–33.49	0.200	2	(1.2)	0		(0.0)	-	-	-	5	(3.1)	1	(1.0)	0.29	0.03–3.04	0.303
Common cold (*n =* 1952)	52	(4.5)	20	(2.5)	0.59	0.37–0.92	**0.021**	26	(2.3)	8		(1.0)	0.45	0.20–1.01	0.054	31	(2.7)	8	(1.0)	0.39	0.18–0.84	**0.016**

**NOTES:** J01C *=* β-lactam antibacterials, penicillins; J01D *=* other β-lactam antibacterials; J01F *=* macrolides; ^¶^ exacerbation of chronic obstructive pulmonary disease/asthma/bronchitis. Statistically significant values are marked in bold.

**Table 6:** Antibiotic prescribing by antibiotic class, pre- and post-intervention.

Antibiotic Class	N (%)		Pre-Intervention		Post-Intervention		OR	95% CI	*p*-Value
			*n* (%)		*n* (%)				
J01A tetracyclines	29	(1.3)	9	(0.7)	20	(2.2)	3.45	1.55-7.69	**0.002**
J01C β-lactam antibacterials, penicillins	1064	(49.5)	590	(46.8)	474	(53.3)	1.20	1.02-1.42	**0.032**
J01D other β-lactam antibacterials	359	(16.7)	242	(19.2)	117	(13.1)	0.61	0.48-0.78	**0.000**
J01F macrolides	633	(29.4)	375	(29.8)	258	(29.0)	1.05	0.87-1.27	0.617
J01M quinolones	74	(3.4)	50	(4.0)	24	(2.7)	0.75	0.45-1.24	0.266
TOTAL antibiotic prescriptions *	**2150**	**(100.00)**	**1260**	(100.0)	890	(100.0)	0.88	0.80-0.98	**0.016**

**NOTES:** * defined as an antibiotic prescription—immediate or delayed—of oral antibiotics issued for an acute respiratory tract complaint during an in-person consultation, irrespective of the number of antibiotics given. Statistically significant values are marked in bold.

**Table 7:** Key process indicators for all 5 intervention components.

Intervention Components *	Process Indicators				
Educational sessions		Session 1	Session 2	Session 3	Session 4
	Duration of session, hrs	2	2	2	2
	Attendance in person, %	50	54	38	42
	Attendance online (live), %	4	8	8	8
	Attendance online (recorded), %	13	8	17	8
	Attendance (total), %	67	71	63	58
Waiting room posters	No. of poster sets printed and disseminated	41			
Patient booklets	No. of booklets printed and disseminated	8600			
National antibiotic guidelines	No. of guidelines printed and disseminated	24			
DAP pads ^#^	No. of DAPs printed (total) and disseminated	5700			
	No. of DAP pads disseminated	190			

**NOTES:** DAP-delayed antibiotic prescription; * total number of participating general practitioners *=* 24; ^#^ 30 DAP prescriptions/pad.

## Data Availability

Relevant data for this study are presented in the tables. Any further data requests are available upon request from the corresponding author.

## Supplementary Materials

The following are available online at https://www.mdpi.com/article/10.3390/antibiotics10040371/s1, Suppl. File 1: Six-page patient booklet on how to manage respiratory tract infections appropriately (English version); Suppl. File 2: Set of four pre-tested posters used during the intervention; Suppl. File 3: Delayed antibiotic prescription pads (front and back) coupled with patient information on respiratory tract infections and appropriate antibiotic use.
