# Supplementary material for: Impact of a Social Marketing Intervention on General Practitioners’ Antibiotic Prescribing Practices for Acute Respiratory Tract Complaints in Malta
Source: Antibiotics (Basel). 2021 Mar 31;10(4):371. doi: 10.3390/antibiotics10040371 (PMC8066227; doi:10.3390/antibiotics10040371)
Supplement: Supplementary file 1 [file antibiotics-10-00371-s001.pdf]

antibiotics

Supplementary Materials File

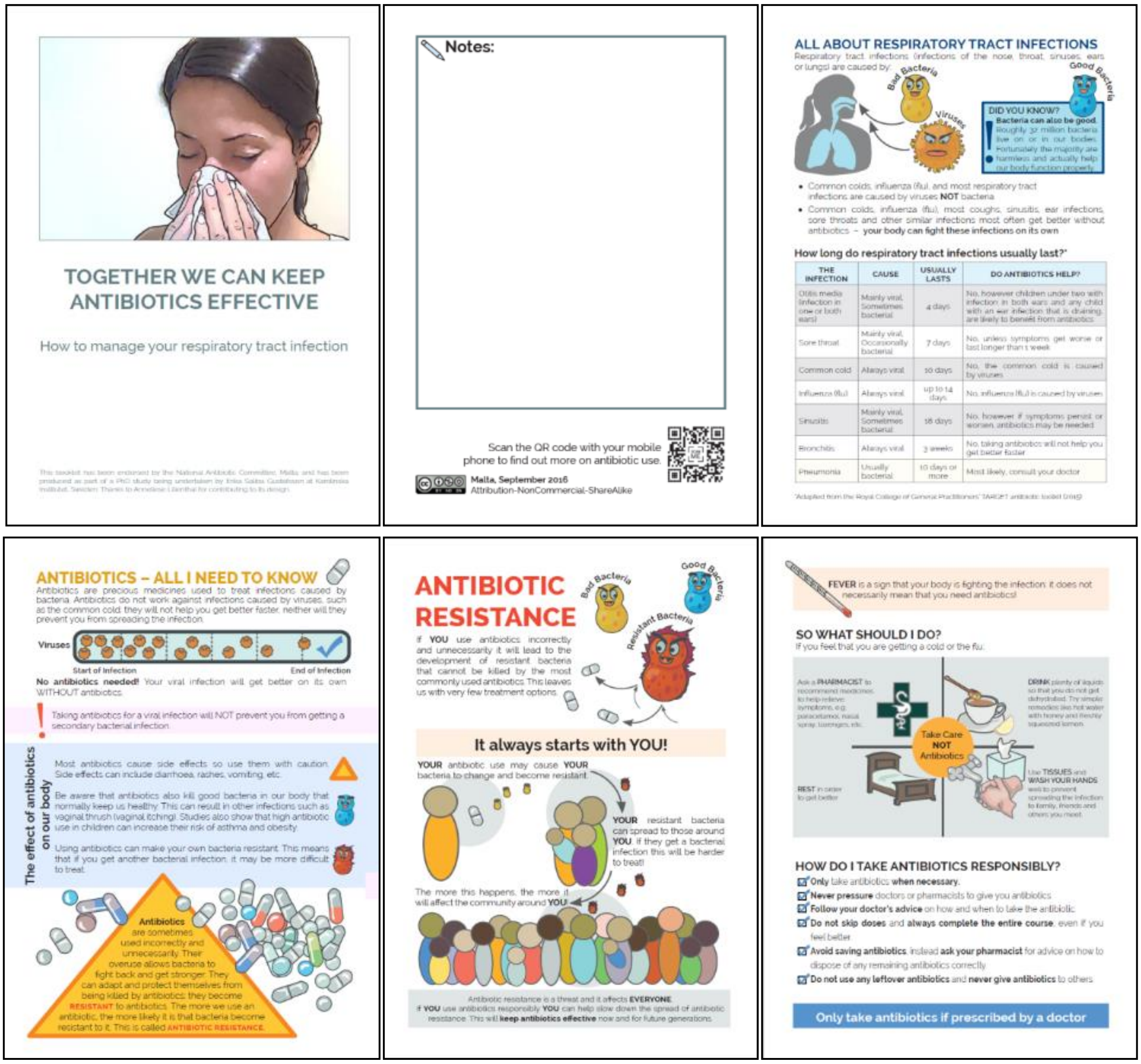

Figure S1. Six-page patient booklet on how to manage respiratory tract infections appropriately (English version).

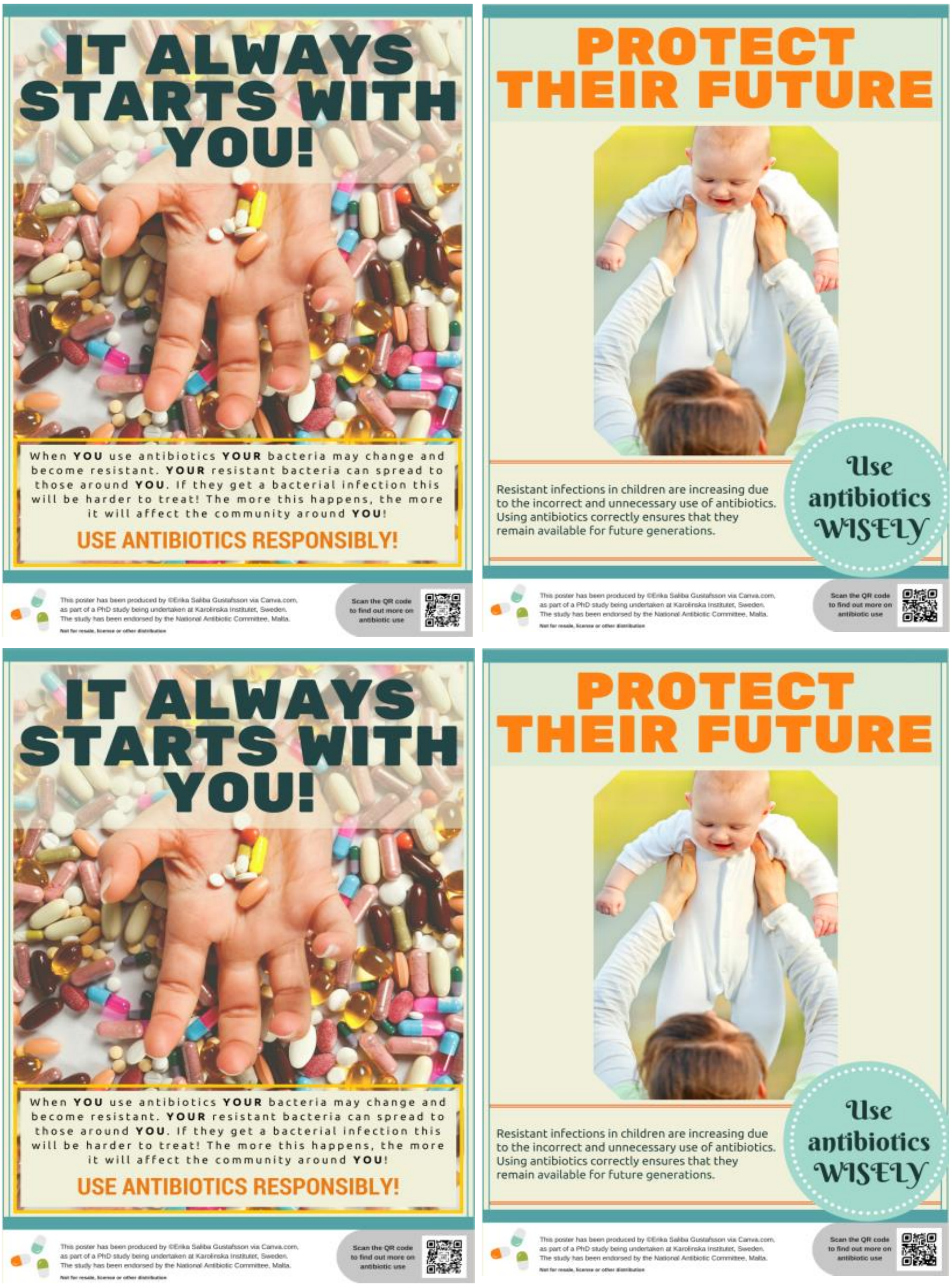

Figure S2. Set of four pre-tested posters used during the intervention.

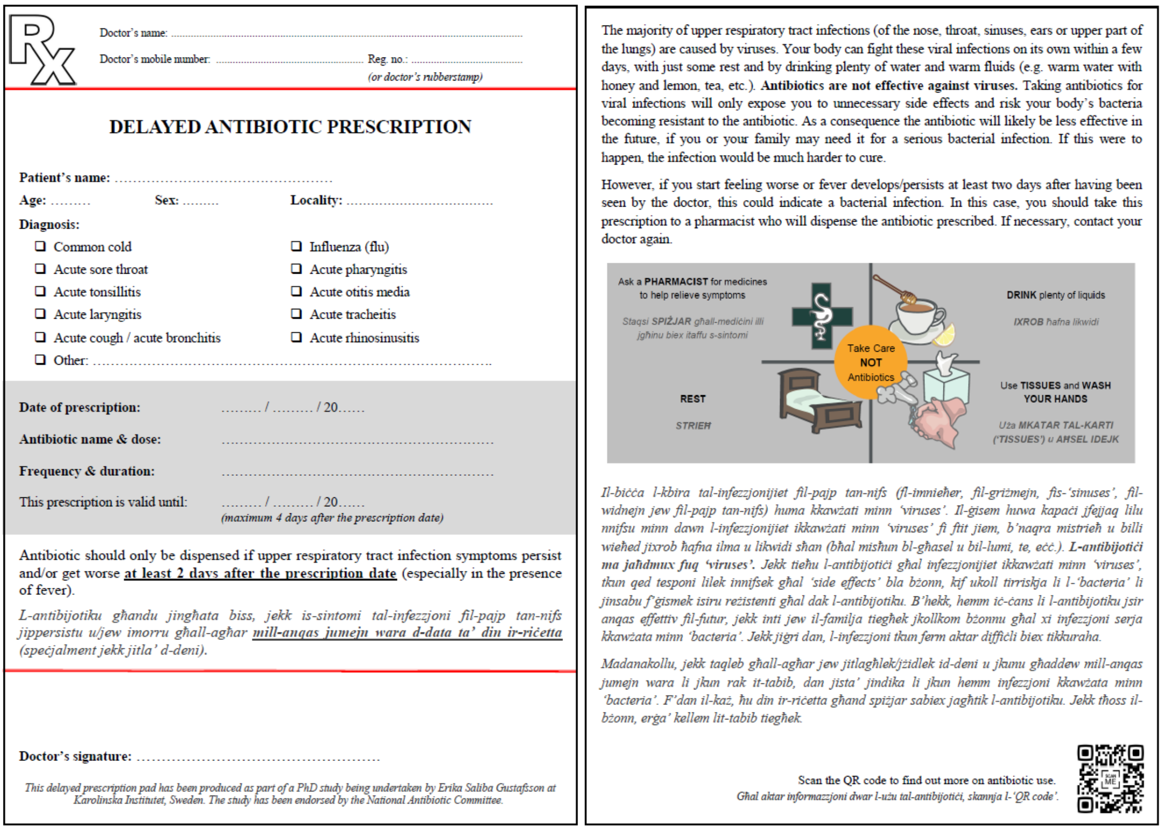

Rx

Doctor's name: ....

Doctor's mobile number: .... Reg. no.: ....
(or doctor's rubberstamp)

DELAYED ANTIBIOTIC PRESCRIPTION

Patient's name: ....

Age: .... **Sex:** .... **Locality:** ....

Diagnosis:

- ❑ Common cold
- ❑ Acute sore throat
- ❑ Acute tonsillitis
- ❑ Acute laryngitis
- ❑ Acute cough / acute bronchitis
- ❑ Other: ....
- ❑ Influenza (flu)
- ❑ Acute pharyngitis
- ❑ Acute otitis media
- ❑ Acute tracheitis
- ❑ Acute rhinosinusitis

Date of prescription: .... / .... / 20......

Antibiotic name & dose: ....

Frequency & duration: ....

This prescription is valid until: .... / .... / 20......
(maximum 4 days after the prescription date)

Antibiotic should only be dispensed if upper respiratory tract infection symptoms persist and/or get worse **at least 2 days after the prescription date** (especially in the presence of fever).

L-antibijotiku għandu jingħata biss, jekk is-sintomi tal-infezzjoni fil-pajp tan-nifs jippersistu u/jew imorru għall-agħar ***mill-anqas jumejn wara d-data ta' din ir-riċetta*** *(speċjalment jekk jitla' d-deni).*

Doctor's signature: ....

This delayed prescription pad has been produced as part of a PhD study being undertaken by Erika Saliba Gustafsson at Karolinska Institutet, Sweden. The study has been endorsed by the National Antibiotic Committee.

The majority of upper respiratory tract infections (of the nose, throat, sinuses, ears or upper part of the lungs) are caused by viruses. Your body can fight these viral infections on its own within a few days, with just some rest and by drinking plenty of water and warm fluids (e.g. warm water with honey and lemon, tea, etc.). **Antibiotics are not effective against viruses.** Taking antibiotics for viral infections will only expose you to unnecessary side effects and risk your body's bacteria becoming resistant to the antibiotic. As a consequence the antibiotic will likely be less effective in the future, if you or your family may need it for a serious bacterial infection. If this were to happen, the infection would be much harder to cure.

However, if you start feeling worse or fever develops/persists at least two days after having been seen by the doctor, this could indicate a bacterial infection. In this case, you should take this prescription to a pharmacist who will dispense the antibiotic prescribed. If necessary, contact your doctor again.

Il-biċċa l-kbira tal-infezzjonijiet fil-pajp tan-nifs (fl-imnieħer, fil-griżmejn, fis-'sinuses', fil-widnejn jew fil-pajp tan-nifs) huma kkawżati minn 'viruses'. Il-ġisem huwa kapaċi jfejjaq lilu nnifsu minn dawn l-infezzjonijiet ikkawżati minn 'viruses' fi ftit jiem, b'naqra mistrieħ u billi wieħed jixrob ħafna ilma u likwidi sħan (bħal misħun bl-għasel u bil-lumi, te, eċċ.). ***L-antibijotiċi ma jaħdmux fuq 'viruses'.*** *Jekk tieħu l-antibijotiċi għal infezzjonijiet ikkawżati minn 'viruses', tkun qed tesponi lilek innifsek għal 'side effects' bla bżonn, kif ukoll tirriskja li l-'bacteria' li jinsabu f'ġismek isiru reżistenti għal dak l-antibijotiku. B'hekk, hemm iċ-ċans li l-antibijotiku jsir anqas effettiv fil-futur, jekk inti jew il-familja tiegħek jkollkom bżonnu għal xi infezzjoni serja kkawżata minn 'bacteria'. Jekk jiġri dan, l-infezzjoni tkun ferm aktar diffiċli biex tikkuraha.*

Madanakollu, jekk taqleb għall-agħar jew jitlagħlek/jżidlek id-deni u jkunu għaddew mill-anqas jumejn wara li jkun rak it-tabib, dan jista' jindika li jkun hemm infezzjoni kkawżata minn 'bacteria'. F'dan il-każ, ħu din ir-riċetta għand spiżjar sabiex jagħtik l-antibijotiku. Jekk tħoss il-bżonn, erġa' kellem lit-tabib tiegħek.

Scan the QR code to find out more on antibiotic use.
Għal aktar informazzjoni dwar l-użu tal-antibijotiċi, skannja l-'QR code'.

Figure S3. Delayed antibiotic prescription pads (front and back) coupled with patient information on respiratory tract infections and appropriate antibiotic use.
